# Self‐Splittable Transcytosis Nanoraspberry for NIR‐II Photo‐Immunometabolic Cancer Therapy in Deep Tumor Tissue

**DOI:** 10.1002/advs.202204067

**Published:** 2022-09-08

**Authors:** Li Wang, Wei Jiang, Yanhong Su, Meixiao Zhan, Shaojun Peng, Hang Liu, Ligong Lu

**Affiliations:** ^1^ Guangdong Provincial Key Laboratory of Tumor Interventional Diagnosis and Treatment Zhuhai People's Hospital (Zhuhai Hospital Affiliated with Jinan University) Zhuhai Guangdong 519000 P. R. China; ^2^ Department of Radiology The First Affiliated Hospital of USTC University of Science and Technology of China Hefei Anhui 230001 P. R. China

**Keywords:** immunogenic cell death, immunosuppression, NIR‐II photo‐immunometabolic cancer therapy, transcytosis, tumor penetration

## Abstract

Cancer photo‐immunotherapy (CPIT) as an ideal strategy can rapidly release hostile signals by appropriate dosage of focal laser irradiation to unmask primary tumor immunogenicity and can activate adaptive immunity to control distant metastases. However, many factors, including disordered immunometabolism, poor penetration of photothermal agents and immuno‐regulators, inadequate laser penetration into the deep tumor region, restrict the therapeutic outcomes of CPIT. Here, a second near‐infrared window (NIR‐II) photo‐immunometabolic cancer therapy (PICT) by a programmed raspberry‐structured nanoadjuvant (PRN^MT^) is presented that can potentiates efficient immunogenic cell death (ICD) in deep tumor tissue and alleviates immunometabolic disorder. The PRN^MT^ is architected through self‐assembly of indoleamine 2,3‐dioxygenase 1 (IDO‐1) inhibitor modified small‐sized CuS nanoparticles (CuS_5_) and tumor microenvironment (TME) responsive cationized polymeric matrix. The TME can trigger the splitting and surface cationization of PRN^MT^ into small cationized CuS_5_ that feature high transcytosis potential and TME immunometabolic regulation. Upon NIR‐II irradiation, CuS_5_ induce homogeneous ICD and release immunometabolic regulator in deep tumor tissues, which ameliorates IDO‐1 mediated immunometabolic disorder and further suppresses regulatory T cells infiltration. PRN^MT^ mediated PICT effectively delays the primary murine mammary carcinoma 4T1 tumor growth and inhibits the lethal pulmonary metastasis in combination with programmed cell death protein 1 (PD1) blockade.

## Graphical abstract

A NIR‐II photo‐immunometabolic cancer therapy by a programmed raspberry‐structured nanoadjuvant (PRN^MT^) is developed to potentiate immunogenic cell death in deep tumor tissue and alleviate immunometabolic disorders. The PRN^MT^ can split into small cationized CuS_5_ that feature high transcytosis potential and TME immunometabolic regulation in tumors. PRN^MT^ under NIR‐II irradiation can reduce breast primary 4T1 tumor burden and inhibit lung metastasis.

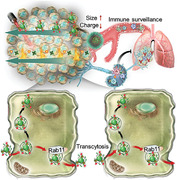

## 1 Introduction

Immunotherapy has been demonstrated as one of the most effective and promising approaches to prolong remissions of cancer in clinical trials.^[^ ^1^, ^2^, ^3^, ^4^ ^]^ Due to the initial striking results of its application among a subset of patients with certain cancers, various combination treatments with immunotherapy have been developed to achieve more efficient performance.^[^ ^5^, ^6^, ^7^, ^8^, ^9^ ^]^ However, immunotherapy fails to exert a valid inhibition of tumor progression due to the limited immunogenicity of tumors and poor infiltration of cytotoxicity T lymphocytes (CTLs). Recently, photo‐immunotherapy has been identified as a promising treatment strategy for many malignant carcinomas.^[^ ^10^, ^11^, ^12^ ^]^ Laser irradiation‐caused hyperthermia can induce immunogenic cell death (ICD) that is associated with the extracellular release of damage‐associated molecular patterns (DAMPs).^[^ ^13^, ^14^, ^15^ ^]^ Antigen presenting cells engulf and process the tumor associated antigen to boost the CTL infiltration and activation for tumoricidal attack.^[^ ^16^, ^17^ ^]^ However, there are several obstacles that impede therapeutic outcomes of photothermal (PTT)‐based immunotherapy. On one hand, poor penetration of photothermal agents fail to induce sufficient immunogenicity in deep tumor tissues due to inhomogeneous DAMPs release, which causes localized tumor regions remaining “cold”.^[^ ^18^, ^19^, ^20^ ^]^ On the other hand, inadequate light penetration cross tissues and sharp heat attenuation in deep tumor tissues are considered to be two important technical hurdles in clinical trials.^[^ ^21^, ^22^, ^23^ ^]^ Thus, enhancing deep and uniform penetration of photothermal agents as well as elevating light penetration depth in tumor tissue for the massive homogeneous production of DAMPs are critical for the effective PTT‐induced immune stimulation.

Moreover, the suppressive metabolic TME (such as acidity, hypoxia, and anomalous tryptophan consumption) generally restricts the efficacy of a CTL‐based antitumor response and weakens the ICD‐induced immune performance.^[^ ^24^, ^25^, ^26^ ^]^ Notably, the massive amount of indoleamine 2,3‐dioxygenase 1 (IDO‐1) in tumors induces an accelerated catabolism of tryptophan, producing abundant kynurenine that severely suppresses the activity of CTLs while inducing their anergy in tumors.^[^ ^27^, ^28^, ^29^ ^]^ In addition, immunosuppressive regulatory T cells (Tregs) benefit from the kynurenine, leading to an impaired antitumor immunity, rapid tumor progression and distant metastasis.^[^ ^30^, ^31^, ^32^, ^33^, ^34^ ^]^ Generally, active recruitment of CTLs toward tumor sites and regulation of immunosuppressive TME as well as mobilization of systemic immune surveillance are demonstrated as effective strategies for the long‐term inhibition of primary tumor growth and metastasis.^[^ ^35^, ^36^, ^37^, ^38^, ^39^ ^]^ Combination treatment of therapeutic methods (include chemotherapy, PTT, and photodynamic therapy) for ICD induction and IDO inhibition can synergistically enhance the therapeutic efficacy of immunotherapy.^[^ ^40^, ^41^, ^42^, ^43^, ^44^, ^45^, ^46^, ^47^ ^]^ However, the regulation of suppressive immunometabolic TME in deep tumor tissue after photothermal‐induced ICD remains challenging.

**Scheme 1:**
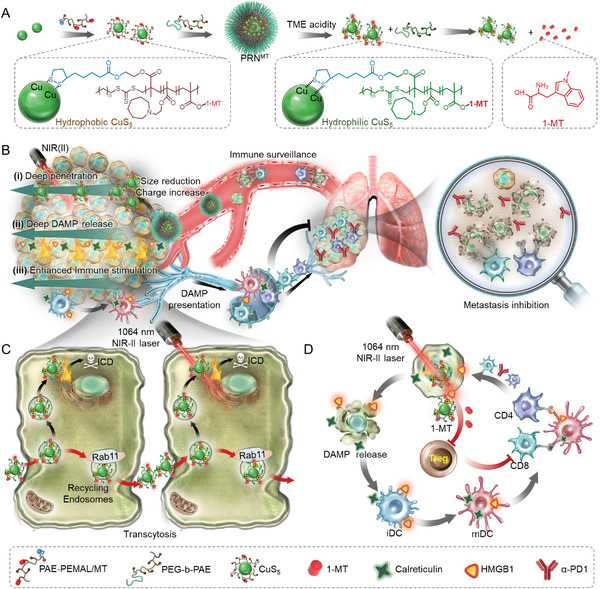
Schematic showing the splitting CuS‐architected raspberry‐structured nanoadjuvant‐mediated NIR‐II photo‐immunometabolic cancer therapy through the induction of ICD induction and IDO inhibition. A) The PRN^MT^ that architected by small size CuS_5_ and TME‐responsive polymer matrix maintains a large size and negative surface charge in physiological microenvironment and splits into small‐sized CuS_5_ with cationized surface when subjecting to acidic TME. B,C) In response to the acidic TME, small‐sized and cationized CuS_5_ release from PRNs^MT^ and penetrate avascular region away from tumor vessels by extracellular diffusion and intracellular transcytosis. Upon NIR‐II irradiation, the released CuS_5_ produce thermal performance in deep region and induce homogenous ICD performance. D) Specifically, tumor cells undergoing ICD promote DAMP emission and activate dendritic cells (DCs), eliciting a potent T cell immune response. In parallel, the loaded 1‐MT on CuS_5_ can block IDO enzyme activity and reverse immunosuppression of CTL responses, leading to the eradication of residual tumors and distant metastasis. Moreover, PRNs^MT^ can delay the growth of distant metastases in combination with immune checkpoint inhibition.

In this study, we presented a NIR‐II photo‐immunometabolic cancer therapy (PICT) by a programmed raspberry‐structured nanoadjuvant (defined as PRN^MT^) with size and charge dual‐transformable abilities as well as immunometabolic regulation in deep tumor tissues (**Scheme** **1**). Specifically, PRNs^MT^ were constructed by IDO inhibitor (D)‐1‐methyltryptophan prodrug (1‐MT) conjugated 5 nm copper sulfide nanoparticles (defined as CuS_5_) with the assistance of a TME responsive polymer matrix. The PRNs^MT^ demonstrated a neutral surface charge and could rapidly dissociate into small‐sized CuS_5_ with a positive surface charge when subjected to the TME condition (pH 6.7). CuS_5_ were rapidly internalized by tumor cells and penetrated deeper tumor tissues via active transcytosis and passive diffusion. Moreover, the strong absorption in the second near‐infrared biowindow (NIR‐II, 950−1350 nm) enabled the CuS_5_ with excellent photothermal performance and generated substantial heat in deep tumor tissues.^[^ ^48^ ^]^ 1‐MT released from PRNs^MT^ effectively inhibited IDO activity and alleviated immunometabolic disorders, which in turn sensitized the PICT. Consequently, PRNs^MT^ significantly retarded primary tumor progression and established a long‐term immunological memory to eradicate distant metastasis.

## 2 Results and Discussion

### 2.1 Synthesis and Characterization of PRNs and PRNs^MT^

**Figure 1:**
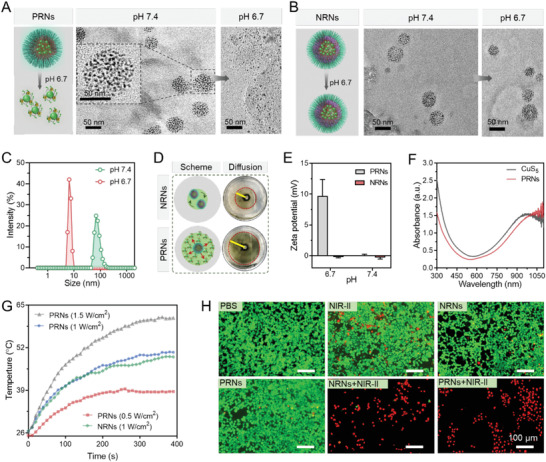
Preparation and characterization of PRNs. TEM images of TME‐responsive PRNs (A) and non‐responsive NRNs (B) at pH 6.7 and pH 7.4, respectively. C) Size transition of PRNs at the acid and neutral pH measured by DLS. D) Images of PRNs diffusion in agarose gel at pH 6.7. E) The zeta potential of PRNs and NRNs at pH 6.7 and 7.4. F) UV–Vis–NIR spectra of PRNs and CuS_5_. G) Photothermal conversion properties of PRNs and NRNs under 1064 nm laser irradiation with varying power densities. H) Calcein‐AM/PI staining by 4T1 cells after pre‐incubated with different formulations with or without laser irradiation (1064 nm, 1 W cm^−2^, 5 min) at pH 6.7 (Scale bar, 100 µm).

To fabricate TME responsive size‐transformable and charge‐reversible PRNs, pH responsive polymer poly(2‐azepane ethyl methacrylate)‐*random*‐poly(ethyl methacrylate of lipoic acid) (PAE‐*r*‐PEMAL) and methoxyl poly(ethylene glycol)‐*block*‐poly(2‐azepane ethyl methacrylate) (mPEG‐*b*‐PAE) were synthesized and are illustrated in Figure S1, Supporting Information. Mono‐dispersed 5 nm CuS nanoparticles were synthesized through a seeding growth method as previously reported.^[^ ^49^ ^]^ The resulting CuS nanoparticles were then coated with PAE‐*r*‐PEMAL in a mass ratio of 1:1, which generated CuS@PAE (defined CuS_5_). PRNs were orchestrated by CuS_5_ and PEG‐*b*‐PAE in a mass ratio of 4:1 by a nano‐precipitation method. The non‐responsive raspberry‐structured nanoadjuvants (NRNs) were obtained in a similar procedure with the non‐pH responsive polymer of poly(2‐cyclohexylethyl methacrylate)‐block‐poly (ethyl methacrylate lipoic acid) (PCM‐*r*‐PEMAL) and poly (ethylene glycol)‐*block*‐poly (2‐cyclohexylethyl methacrylate) (mPEG‐*b*‐PCM) (Figure S2, Supporting Information), respectively. In addition, we conjugated 1‐methyl‐tryptophan (1‐MT), a competitive inhibitor of IDO, on PAE‐*r*‐PEMAL by esterification. 1‐MT modified CuS_5_ was obtained by coupling CuS_5_ nanoparticles with PAE‐*r*‐PEMAL/MT through lipoic acid. The chemical structures of 1‐MT‐based polymers and other related polymers were identified by ^1^H nuclear magnetic resonance (^1^H NMR) spectra (Figures S3–S7, Supporting Information). Both PRNs and NRNs demonstrated similar sizes and morphologies observed by transmission electron microscope (TEM) (**Figures** **1**A and 1B). The raspberry structure of the PRNs was highly stable at pH 7.4 (normal tissue) and sharply disintegrated into small‐sized CuS_5_ nanoparticles at pH 6.7 (TME), which was confirmed by dynamic light scattering (DLS) (Figure 1C). By contrast, the size and structure of NRNs did not change in the condition of pH 6.7. An agarose gel was used to evaluate the diffusion of PRNs in response to acidic conditions. The PRNs showed enhanced diffusion due to size reduction (Figure 1D). After protonation of the PAE segments, the zeta potential of the PRNs increased from near 0 to +10 mV, whereas the NRNs stayed near neutral (Figure 1E).

Inspired by the strong absorbance in the NIR‐II region from UV‐Vis‐NIR‐II spectroscopy (Figure 1F), we further evaluated the photothermal transition of PRNs when it was exposed to NIR‐II laser (1064 nm). After 5 min irradiation, the temperature increased from 25 to 50 and 62 °C with a laser power of 1 and 1.5 W cm^−2^ at 100 µg mL^−1^ of CuS, respectively (Figure 1G). It indicated that PRNs were potent photothermal therapeutic agents. The PRNs also demonstrated high photostability after 3 heating–cooling cycles (Figure S8, Supporting Information). The pH responsive dissociation of PRNs did minimal influence on the photothermal conversion efficiency (Figure S9, Supporting Information). According to Equation (1) (Supporting Information, Eq.), heat conversion efficiency (*η*) of the PRNs under 1064 nm laser irradiation was 27.4%. Moreover, the PRNs were stable in pure water even after 4 days’ storage (Figure S10, Supporting Information). After incubation in 10% FBS, the size of PRNs showed a slight increase and stay invariable (Figure S11, Supporting Information). We further evaluated the cumulative release of 1‐MT from PRN^MT^ under irradiation or not. The NIR‐II light irradiation in the first 5 min incubation enhanced the 1‐MT release. The most part of 1‐MT could be released after 12 h incubation with cell lysates (Figure S12, Supporting Information). We further evaluated the cytotoxic effects of PRNs with or without 1064 nm irradiation by MTT and dead/live staining assay. PRNs and NRNs did not show significant cytotoxicity against tumor cells without 1064 nm laser irradiation from MTT results (Figure S13, Supporting Information). However, after irradiation with 1064 nm laser, PRNs and NRNs significantly suppressed cell proliferation, and almost killed all tumor cells at a concentration of 100 µg mL^−1^ CuS (Figure S14, Supporting Information). This PRN‐based photothermal cytotoxicity was further supported by Calcein‐AM/PI staining at pH 6.5 (Figure 1H).

### 2.2 Active Transcytosis of Protonated PRNs in TME

**Figure 2:**
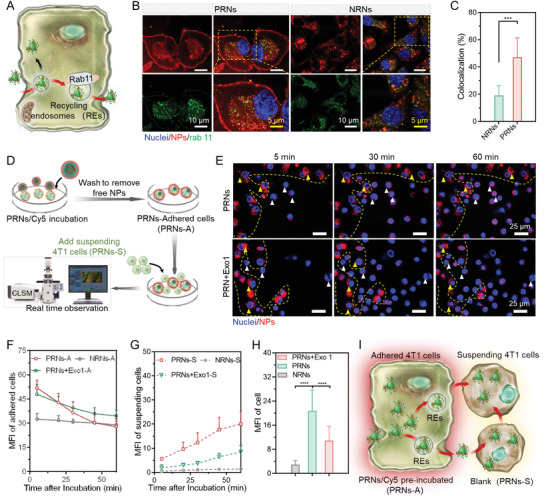
Enhanced transcytosis of PRNs at pH 6.7. A) Schematic showing recycling endosomes transport of intracellular cargos to extracellular space. B) Confocal images showing co‐localization of rab11^+^ recycling endosomes (green) and PRNs (red) at pH 6.7. C) Calculation of the co‐localization index of rab 11^+^ recycling endosomes and PRNs. D,E) Schematic illustration of real‐time monitor and confocal images of active transcytosis of PRNs at pH 6.7. F,G) Time‐dependent MFI changes of PRNs/Cy5 in adhered cells (F) and suspending cells (G) (*n* = 3). H) MFI calculation of the added suspending cells after 60 min incubation with adhered cells (*n* = 12). I) Schematic showing that transcellular transport of PRNs^MT^ from adhered cells to suspending cells. Data are shown as mean ± SD by one‐way analysis of variance (ANOVA) was used. **p < 0.005, ***p < 0.001, ****p < 0.0001.

Due to cationized surface of PRNs after being subjected to an acidic TME, it was anticipated that PRNs would be transported across multilayer cells through active transcytosis. Rab11 is a distinct marker of recycling endosomes that charges dynein‐mediated transport from cytoplasm to extracellular space.^[^ ^50^ ^]^ We thus investigated the distribution of rab11^+^ endosomes and internalized nanoparticles at a pH 6.7 condition (**Figure** **2**A,B). PRNs and NRNs were labeled with Cy5 by conjugating Cy5‐COOH to the PAE‐*r*‐(PHEMA‐PEMAL) for visualization by confocal laser scanning microscopy (CLSM). After incubation for 4 h, signal of Cy5 in PRNs showed an evident membrane absorption and co‐localization with rab11^+^ recycling endosomes (REs). Approximately 45% of PRNs was co‐localized with rab11^+^ REs, which was 2.2‐fold higher than that of the NRN group (Figure 2C).

To further evaluate the transcellular transport of PRNs from one cell to another, PRNs pre‐treated adhered 4T1 cells were co‐incubated with newly added 4T1 cells in a fresh medium for real‐time observation by CLSM (Figure 2D). During incubation, PRNs were transported from adhered cells (inside the yellow dash line) to suspending cells (outside the yellow dash line) quickly (Figure 2E). By calculating the median fluorescence intensity (MFI) of adhered cells (yellow triangles) and suspending cells (white triangles), we found that the fluorescence signal of PRNs faded as time extended in adhered cells, indicating the gradual efflux of PRNs (Figure 2F). Simultaneously, the fluorescence intensity of PRNs in suspending cells went higher as time extended, which suggested that PRNs were transported from adhered cells to suspending cells (Figure 2G). However, NRNs were still trapped in adhered cells even after 60 min of co‐incubation (Figure S15, Supporting Information). After inhibiting the exocytosis of tumor cells by adding Exo1, a cellular vesicular trafficking inhibitor, most of PRNs localized in the suspending cells (Figure 2H). The above results demonstrated that PRNs can be efficiently transcellularly transported during co‐incubation, whereas NRNs failed to be transported due to near‐neutral surface charge (Figure 2I).

### 2.3 Deep Tumor Penetration of PRNs

**Figure 3:**
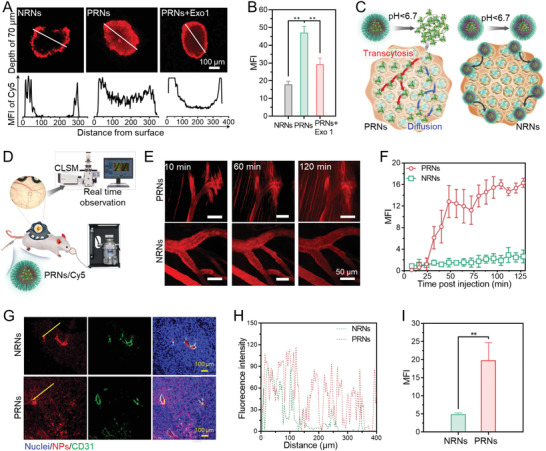
Active transcytosis of PRNs contributes to deeper tumor penetration. A) Confocal images showing the penetration behavior of NRNs and PRNs into MCSs at pH 6.7 and pH 7.4. B) MFI of MCSs calculated by ImageJ. C) Scheme showing PRN penetration in MCSs. PRNs can penetrate into deep region of MCSs through enhanced active transcytosis and passive diffusion. D) Scheme showing the process of real‐time observation of PRN microdistribution in tumors. E) In vivo real‐time observation of the microdistribution of PRNs and NRNs in 4T1 tumors after intravenous (i.v.) administration. F) Time‐dependent MFI changes of PRNs in the extravascular tumor tissues. G) Confocal images of typical tumor tissue sections after i.v. injection of PRNs for 12 h. The tumor vessels were labelled by CD31‐FITC (green) and nuclei were labelled by DAPI (blue). H) Profiles showing the fluorescence intensity along the solid yellow lines on the panel (G). I) MFI of Cy5 in tumor tissues after treatment of PRNs. Data are shown as mean ± SD by one‐way analysis of variance (ANOVA) or unpaired two‐tailed Student's *t*‐tests were used (*n* = 3). **p < 0.005, ***p < 0.001, ****p < 0.0001.

The extracellular matrix, high interstitial fluid pressure and tight junction between tumor cells have been reported as the substantial barriers for impeding the penetration of nanoparticles into deep tumor sites away from the vessels.^[^ ^51^ ^]^ Thus, we further employed a 4T1 multi‐cell spheroid (MCS) model to mimic the solid tumor morphology and TME. After co‐incubation for 4 h at pH 6.7, the NRNs failed to penetrate into MCSs, as evidenced by the distribution of a mostly Cy5 signal around the periphery of MCSs (**Figure** **3**A,B). However, PRN‐treated MCSs showed a strong and homogeneous fluorescent signal even at a depth of 70 µm from the surface of the MCSs, indicating that PRNs with active transcytosis abilities could penetrate into deeper tissues. After inhibiting the exocytosis of MCSs by Exo1 at pH 6.7, PRNs failed to penetrate the core of MCSs, which further validated the contribution of PRN‐based transcytosis to tumor penetration (Figure 3C).

We next investigated the penetration behavior of PRNs in vivo by observing their micro‐distribution in a 4T1 tumor model using real‐time monitoring by CLSM (Figure 3D). As expected, the NRNs were unable to penetrate avascular deep tumor tissues even at 110 min post intravenous (i.v.) injection (Figure 3E). In contrast, PRNs gradually extravasated from tumor vessels and penetrated into a deep tumor interstitial space within 120 min, indicating their penetration superiority in vivo (Figure 3F). After 12 h post‐i.v. injection of PRNs, the tumors were excised for frozen sections and tumor vessels were labelled with CD31‐FITC. We observed that NRNs were trapped around the blood vessels and unable to penetrate into deep tumor tissues. Whereas PRNs showed deeper penetration, distributed homogeneously and faded as the distance from vessels increased (Figure 3G). A corresponding median fluorescence intensity (MFI) of Cy5 demonstrated that the tumor accumulation of PRNs was ≈3.5‐fold higher than that of NRNs (Figure 3H,I). Collectively, these results suggest that the detached CuS_5_ from TME responsive PRNs with small size and surface cationization can efficiently penetrate into deep tumor tissue via extracellular diffusion and intracellular transcytosis.

### 2.4 Enhanced ICD Performance of PRNs in Deep Tumor Tissues

**Figure 4:**
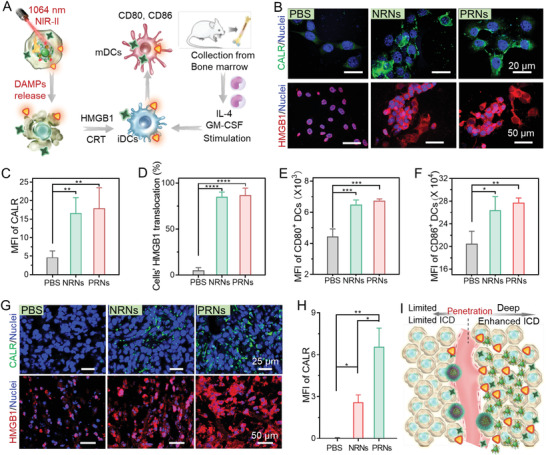
PRNs enhanced ICD performance and subsequent immune stimulation in deep tumor tissue. A) Scheme showing that the tumor cells undergoing ICD after PRNs plus NIR‐II laser irradiation treatment release immunogenic DAMPs and promote DC maturation. B) Confocal images showing the exposure of CALR and the translocation of HMGB1 in 4T1 cells at 4 and 20 h after NIR‐II laser irradiation. MFI of C) CALR exposure and D) HMGB1 translocation from nuclei to cytoplasm in 4T1 tumor cells. E,F) Flow cytometry analysis (FACS) of activation of purified murine bone marrow derived dendritic cells (BMDCs) after culturing with dying 4T1 cells for 12 h. Expression of E) CD80 and F) CD86 on BMDCs. Data are shown as mean ± SD (*n* = 3). G) Immunofluorescence staining of CALR exposure and HMGB1 release in 4T1 tumors at 4 and 20 h post NIR‐II laser irradiation. H) Corresponding quantifications of the MFI of CALR. I) The PRNs plus NIR‐II laser irradiation can induce massive release of DAMPs in the deeper depth of 4T1 tumors and induce stronger DC activation. Data are shown as mean ± SD by one‐way analysis of variance (ANOVA) or unpaired two‐tailed Student's *t*‐tests were used (*n* = 3). **p < 0.005, ***p < 0.001, ****p < 0.0001.

DAMPs have been identified as an important immunogenic characteristic of ICD.^[^ ^52^ ^]^ Encouraged by the excellent photothermal effects upon NIR‐II light irradiation, we next investigated whether PTT performance of PRNs could induce the release/exposure of DAMPs from dying cells. Among the DAMPs, surface exposed endoplasmic reticulum luminal chaperone calreticulin (CALR) and high mobility group box 1 protein (HMGB1) serve as potent immunostimulatory molecules that recruit and cause the maturity of dendritic cells (DCs) for adaptive immunity activation.^[^ ^53^ ^]^ Thus, we investigated the CALR exposure and HMGB1 translocation after treatment with PRNs plus NIR‐II light irradiation (**Figure** **4**A). After pretreatment with PRNs or NRNs plus 1064 nm light irradiation (1 W cm^−2^) for 4 h, 4T1 cells showed massive CALR exposure compared to the control groups (Figure 4B,C). For the untreated cells, HMGB1 was mostly located in the nuclei. In contrast, significant translocations of HMGB1 from nuclei to the extracellular milieu were observed in the cells following both PRNs and NRNs plus 1064 nm light irradiation treatments (Figure 4B,D). Collectively, PRNs plus NIR‐II light irradiation treatment could be a potent ICD inducer and efficiently trigger the emission of DAMPs in dying cells.

Upon release from dying cells, these DAMPs then bind to cognate receptors on DCs, leading the DCs to acquire a mature phenotype that activates antigen presentation and induces the activation of T cell response.^[^ ^54^ ^]^ To investigate DAMP‐induced DC maturation by PRNs plus NIR‐II irradiation, we extracted and propagated murine bone marrow‐derived dendritic cells (BMDCs) from mouse bone marrow in the stimulation of mouse granulocyte/macrophage colony‐stimulating factor and interleukin‐4 as typical DCs. After PRN plus 1064 nm light irradiation treatment for 12 h, dying 4T1 cells were further incubated with BMDCs for 12 h and analyzed using flow cytometry. The expression of co‐stimulator molecules CD80 and CD86 on the surface of BMDCs represented the level of DC maturation (Figure S16, Supporting Information). Both PRNs‐ and NRNs‐treated tumor cells caused a strong upregulation of co‐stimulator molecules compared to control group (Figure 4E,F). Thus, released DAMPs from PRNs plus 1064 nm light irradiation‐treated 4T1 cells could effectively promote DC maturation and activation. Inspired by the excellent tumor penetration of PRNs, we further evaluated the CALR exposure and HMGB1 translocation in PRNs plus 1064 nm light irradiation‐treated tumors. Compared to the control tumor, NRNs plus 1064 nm light irradiation treated tumors generated moderate CALR exposure. Notably, PRNs plus NIR‐II irradiation treatment induced stronger and more uniform CALR exposure in 4T1 tumors than that of the NRNs (Figure 4G,H). Similarly, HMGB1 staining showed that PRNs caused a stronger HMGB1 translocation than that of NRNs under NIR‐II irradiation (Figure S17, Supporting Information). These results indicated that PRNs with high transcytosis and uniform penetration generated stronger ICD performance than that of NRNs, which in turn cause stronger immune stimulation under NIR‐II light irradiation (Figure 4I).

### 2.5 Tumor Ablation and Immune System Activation of PRN^MT^ Under NIR‐II Irradiation

**Figure 5:**
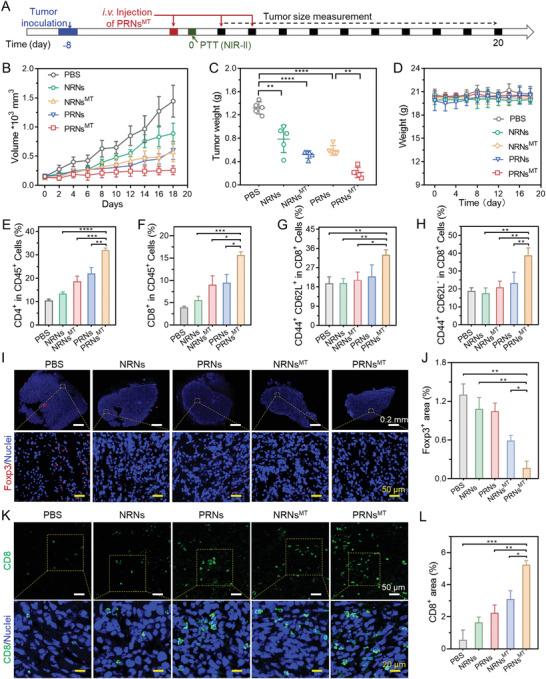
Effective inhibition on established subcutaneous 4T1 tumor model by P RN^MT^ plus NIR‐II laser irradiation. A) Schematic showing the process of antitumor treatments. Tumor cells were inoculated 8 days before injecting therapeutic agents. The tumor volume was measured every 2 days (DOSE of 20 mg CuS per kilogram mouse weight, laser power was 1 W cm^−2^). Data are shown as mean ± SD (*n* = 5), *p < 0.05, **p < 0.005, ***p < 0.001. B) Tumor volume evolution during the treatments. C) Weights of tumor excised at the end of therapy. D) Mice weight changes during the treatment. E–H) After 18 days, the mice were sacrificed and spleens were harvested, and the percentages of E) CD4^+^ T cells, F) CD8^+^ T cells, G) CD62L^+^ CD4^+^ T cells, H) CD62L^+^ CD8^+^ T cells were measured by flow cytometry (*n* = 6). Immunofluorescence staining of Foxp3^+^ (I) and CD8^+^ (K) T cells. Quantifications of Foxp3^+^ (J) and CD8^+^ (L) area in panels (I) and (K). Data are shown as mean ± SD by one‐way analysis of variance (ANOVA) or two‐way ANOVA with Tukey's multiple comparisons test (*n* = 5). **p < 0.005, ***p < 0.001, ****p < 0.0001.

Due to the reprogrammed metabolism of tumor cells in TME, tumor‐infiltrating cytotoxic immune cells typically suffer abnormal metabolic stress, eliciting impaired antitumor immune responses.^[^ ^55^ ^]^ Overexpressed IDO in tumor cells accelerates the catalyzed consumption of tryptophan to kynurenine, which promotes the development of Tregs that impede CTL function.^[^ ^29^ ^]^ To reverse the suppressive immunometabolic TME induced by overexpressed IDO and enhance the activity of CTL, we prepared 1‐MT loaded programmed raspberry‐like nanoadjuvants (PRNs^MT^ and NRNs^MT^) through conjugation 1‐MT to PAE*‐r*‐PEMAL or PCM‐*r*‐PEMAL by esterification. The loaded 1‐MT in PRNs^MT^ was expected to decrease the consumption of tryptophan as well as kynurenine production. We evaluated the photothermal performance in tumor sites and antitumor efficacy of PRNs^MT^ in vivo. The Balb/C mice bearing 4T1 tumors were i.v. injected with PRNs^MT^ (dose of 20 mg CuS per kilogram mouse weight), following local 1064 nm laser irradiation (laser power of 1 W cm^−2^) in tumors at 12 h post‐injections. The temperature of the PRN^MT^‐treated tumor gradually increased to 47 °C under 1064 nm laser irradiation higher than that of the NRNs^MT^ group (Figure S18, Supporting Information), which might be due to the deeper tumor penetration of CuS_5_ released from PRNs^MT^. The antitumor efficacy of PRN^MT^ was further evaluated in 4T1 tumor models. After the 4T1 tumor volume reached 70–100 mm^3^, PBSs, NRNs, NRNs^MT^, PRNs and PRNs^MT^ were i.v. administrated to the mice, respectively (**Figure** **5**A). After 12 h, the 1064 nm laser (1 W cm^−2^) was applied to irradiate tumors at 47 °C for 5 min. The NRNs plus NIR‐II laser irradiation treatment generated weak tumor inhibition (≈38% inhibition) compared to control groups (Figure 5B,C). NRNs^MT^ plus NIR‐II laser irradiation treatment exerted a moderate suppression effect on tumor development (≈56% tumor growth inhibition). Due to the enhanced penetration of PRNs and elevated hyperthermia efficacy, PRNs plus NIR‐II laser irradiation treatment alone delayed tumor development but failed to produce long lasting inhibition after 18 days (≈58% tumor growth inhibition). Notably, PRNs^MT^ plus NIR‐II laser irradiation significantly inhibited tumor growth by ≈83% tumor growth inhibition. The body weight changes and tumor images reflected the high biocompatibility and enhanced antitumor efficacy of PRNs^MT^ plus NIR‐II laser irradiation (Figure 5D and Figure S19, Supporting Information).

To verify the enhanced antitumor immune response induced by PRN^MT^‐mediated ICD induction and IDO blockade, we further measured the immune cells of spleens and tumors from the treated mice via flow cytometry and immunofluorescence staining, respectively. NRNs plus NIR‐II laser irradiation caused moderate elevation of CD4^+^ and CD8^+^ T cells compared to the PBS group. The PRNs group showed higher elevation of CD4^+^ and CD8^+^ T cells than that of the NRNs group after NIR‐II light irradiation (Figures 5E and 5F). The enhanced infiltration of CTLs could be attributed to massive release of DAMPs and immune stimulus. Both attributable to the IDO blockade, PRNs^MT^ and NRNs^MT^ treatments showed higher content of CTL infiltration than PRNs and NRNs, respectively. In addition, the populations of central memory CD4^+^ T cells (T_CM_ in CD4^+^ and CD8^+^) in PRNs^MT^‐treated mice were significantly enhanced, which might elicit immunological memory effects due to the enhanced ICD performance and CTL activation (Figures 5G and 5H). Moreover, the distribution of Foxp3^+^ Treg and CD8^+^ T cells in tumors was investigated by immunofluorescence staining. Consistent with flow cytometry results, intratumor infiltration of Tregs in NRNs^MT^ and PRNs^MT^ plus NIR‐II laser irradiation group was decreased by 55% and 82% compared to the PBS group, respectively (Figure 5I,J). Moreover, the frequency of CD8^+^ T cells in the PRNs^MT^ plus NIR‐II irradiation group was shown to be 1.7‐fold higher than NRNs^MT^ plus NIR‐II irradiation and 2.5‐fold higher than PRNs plus NIR‐II irradiation (Figure 5K,L). Collectively, the above results reveal that PRNs^MT^ plus NIR‐II phototherapy effectively reversed the immune tolerance in primary tumors and established systemic antitumor memory by enhanced ICD induction and effective IDO pathway blockage in deep tumor tissues.

### 2.6 Efficient Inhibition of Tumor Distant Metastases by PRNs^MT^ Combined with *α*‐PD1

**Figure 6:**
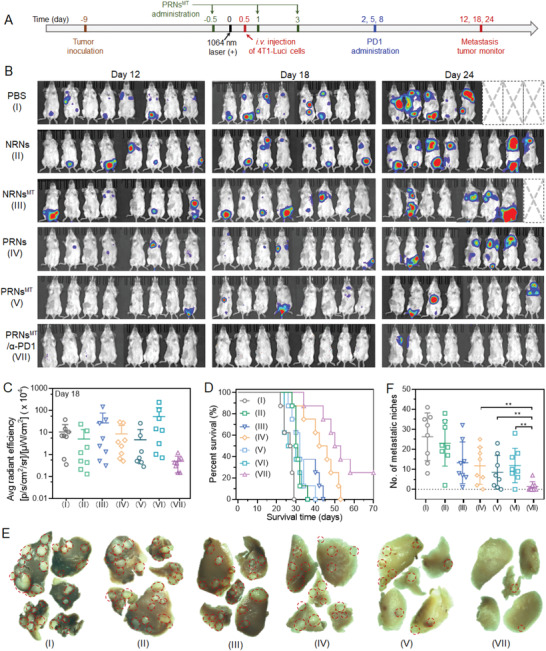
PRNs^MT^ plus NIR‐II irradiation synergizes with *α*‐PD1 blockade therapy and prevents tumor metastasis. A) Subcutaneous 4T1 tumor model was established 8 days before treatment. The mice were treated with different formulations, following with NIR‐II light irradiation to eliminate the primary tumors on the flanks. To facilitate the monitor of metastasis, living 4T1 cells stably expressing luciferase activity (Luc‐4T1) were i.v. injected into the Balb/c mice. B) The distribution of Luc‐4T1 cells in mice were tracked by bioluminescence images on 12, 18, and 24 days after Luc‐4T1 cell injection. C) Corresponding quantifications of tumor bioluminescence on day 24. D) Morbidity‐free survival of mice in response to different treatments (*n* = 8). E) Representative photographs of lung lobes as viewed by stereo microscope (the red circles indicated metastatic nodules). F) Calculation of the number of metastatic niches after different treatments (*n* = 8). Data are shown as mean ± SD by one‐way analysis of variance (ANOVA) or two‐way ANOVA with Tukey's multiple comparisons test (n = 5). **p < 0.005, ***p < 0.001, ****p < 0.0001.

Encouraged by massive CD8^+^ T cell infiltration and an enhanced T_CM_ level, we next evaluated whether PRNs^MT^ combined with an *α*‐PD1 immune checkpoint inhibitors treatment could suppress the lethal pulmonary metastasis of 4T1 tumors. 4T1 cells stably expressed firefly luciferase (Luc‐4T1) were applied to track the metastasis development in mouse bodies. After irradiating subcutaneous primary 4T1 tumors with NIR‐II laser, Luc‐4T1 tumor cells were i.v. injected into mice for visualizing metastatic niche distribution (**Figure** **6**A). In vivo bioluminescence images of mice were captured on days 12, 18, and 24 after i.v. injection with Luc‐4T1 cells. Notably, bioluminescence signals of firefly luciferase were observed in multiple sites of control mice at day 12, indicating the malignant metastasis of Luc‐4T1 cells. NRNs plus NIR‐II laser irradiation treatment failed to suppress the tumor metastasis. NRN^MT^ plus NIR‐II laser irradiation exerted a moderate inhibition in lung metastasis compared to PBS group. PRNs plus laser irradiation treatment delayed metastasis in the first 18 days, whereas rapid metastasis happened afterward. In contrast, PRNs^MT^ plus NIR‐II laser irradiation treatment demonstrated more pronounced inhibition effect on the aggressive tumor metastasis than PRNs plus NIR‐II laser irradiation treatment (Figure 6B,C and Figures S20 and S21, Supporting Information). Moreover, PRNs^MT^ plus NIR‐II laser irradiation in combination with *α*‐PD1 effectively restricted lung metastasis as well as primary tumor growth (Figure S22, Supporting Information). Notably, ≈25% of mice treated with the combination of *α*‐PD1 and PRNs^MT^ plus NIR‐II irradiation survived more than 70 days, while mice that received other regimes all died within 29–54 days during the course of therapy (Figure 6D). When the primary tumor volumes reached 2000 mm^3^, the mice were sacrificed, and the lungs were excised for observation. Large amounts of tumor nodules were observed in PBS‐treated lung lobes while PRNs^MT^ treated mice showed fewer tumor nodules. The number of lung lobes of the combination therapy of *α*‐PD1 and PRNs^MT^ plus irradiation decreased markedly compared to other groups (Figure 6E and Figure S23, Supporting Information). Collectively, our results demonstrated that the PRNs^MT^ synergized with *α*‐PD1 blocker therapy and provided a promising suppressive effect on both primary tumor growth and metastasis, accompanied by long‐term survival as well.

## 3 Conclusion

In summary, a PICT by a TME responsive transcytosis nanoadjuvant (PRN^MT^) loaded with immunometabolic regulator was developed. PRNs^MT^ featured strong absorbance in the NIR‐II window and active transcytosis potential. Specifically, enhanced penetration of PRNs^MT^ under NIR‐II irradiation induced stronger photothermal performance and massive tumor‐associated antigen exposure in deep tumor tissues, which stimulated DC maturation and subsequent immune system activation compared to non‐transcytosis NRNs. The loaded 1‐MT in PRNs^MT^ ameliorated the suppressive immunometabolic TME, eliciting increased CTL infiltration while significantly attenuating Treg infiltration. A combination of *α*‐PD1 and PRNs^MT^‐based PICT effectively inhibited lung metastasis and elevated the survival time of 4T1 tumor‐bearing mice. Our study provided an effective strategy to improve PICT outcomes of primary and metastatic tumors.

## Conflict of Interest

The authors declare no conflict of interest.

## Supporting information

Supporting Information

## Data Availability

The data that support the findings of this study are available from the corresponding author upon reasonable request.
