# Supplementary material for: Self‐Splittable Transcytosis Nanoraspberry for NIR‐II Photo‐Immunometabolic Cancer Therapy in Deep Tumor Tissue
Source: Adv Sci (Weinh). 2022 Sep 8;9(32):2204067. doi: 10.1002/advs.202204067 (PMC9661837; doi:10.1002/advs.202204067)
Supplement: Supplementary file 1 — Supporting Information [file ADVS-9-2204067-s001.pdf]

# Supporting Information

**Title Self-Splittable Transcytosis Nanoraspberry for NIR-II Photo-Immunometabolic Cancer Therapy in Deep Tumor Tissue**

*Li Wang, Wei Jiang, Yanhong Su, Meixiao Zhan, Shaojun Peng,* Hang Liu,* and Ligong Lu**

Dr. L. Wang, Dr. Y. Su, Prof. M. Zhan, Dr. S. Peng, Prof. L. Lu
Guangdong Provincial Key Laboratory of Tumor Interventional Diagnosis and Treatment
Zhuhai People's Hospital (Zhuhai Hospital Affiliated with Jinan University)
Zhuhai, 519000, Guangdong, China
*E-mail: shaojunpeng@ext.jnu.edu.cn
*E-mail: luligong1969@jnu.edu.cn

Dr. L. Wang, Dr. W. Jiang, Dr. H. Liu
Department of Radiology
The First Affiliated Hospital of USTC
University of Science and Technology of China
Hefei, 230001, Anhui, China
*E-mail: hangliu@ustc.edu.cn

**Materials**

*D, L*-lipoic acid (LA), azobisisobutyronitrile (AIBN), methoxyl poly(ethylene glycol) (M*n* 5,000), 2-hydroxyethyl methacrylate, copper acetylacetonate, oleylamine (OAm), 2-cyano-2-propyl dodecyl trithiocarbonate (CPDT) were purchased from Sigma-Aldrich (Saint Louis, MO). Sulfur was purchased from Sinopharm Chemical Reagent Co., Ltd, (Shanghai, China). 1-Cyclohexylethanol, 2-(azepan-1-yl) ethanol, and methacryloyl chloride were purchased from Energy Chemical (Shanghai, China). 1-(3-Dimethylaminopropyl)-3-ethylcarbodiimide hydrochloride (EDC·HCl), 1-methyl-D-tryptophan (1-MT) and *N*-hydroxysuccinimide (NHS) were purchased from Aladdin Reagent Co. Ltd. (Shanghai, China). 3-(4, 5-Dimethylthiazol-2-yl)-2, 5-diphenyltetra-zolium bromide (MTT) was purchased from Sangon Biotech (Shanghai, China). Water was purified by using a Milli-Q purification system (Millipore, Bedford, MA). Other chemicals or solvents without mention were analytical grade and used as received. Programmed death-1 monoclonal antibody (α-PD1) was bought from Bioxcell. Antibodies against cell surface markers for flow cytometry assay were obtained from Biolegend (San Diego, CA). Luciferin and Exo 1 were obtained from Abcam (Massachusetts, US). Calcein/PI cell viability/cytotoxicity assay kit was obtained from Beyotime Biotechnology (Shanghai, China).

**Characterizations**

$^1$H NMR spectra were tested on a Bruker AV400 NMR spectrometer (Bruker Corporation, Switzerland) using $CDCl_3$ as solvent. CuS content was determined on an X Series 2 inductively coupled plasma mass spectroscopy (ICP-MS, Thermo Fisher Scientific, Waltham, MA). Size and zeta potential of nanoparticles measurements were detected using a zeta potential analyzer with dynamic light-scattering (DLS, Malvern Instruments Ltd., UK). Morphology of nanoparticles was examined by JEOL-2010 transmission electron microscopy (TEM, JEOL Co. Ltd., Tokyo, Japan) at an accelerating voltage of 120 kV. UV-Vis-NIR absorption spectra was performed on a UV-Vis-NIR spectrophotometer (Agilent Cary 60) with a quartzcuvette of 1 cm optical path length.

**Methods**

**Synthesis of 1-MT-Boc.**

1-MT (400 mg), di-tert-butyl dicarbonate (Boc anhydride, 520 mg) and $NaHCO_3$ (460 mg) were dissolved in a solution containing 20 mL tetrahydrofuran (THF) and 20 mL $H_2O$. The mixture was reacted at 0 °C for 30 min and then stirredat room temperature overnight. 1 N

HCl (20 mL) was added after removing THF by evaporation. The solution was adjusted to pH 1 by crystal precipitation, followed filtration to obtain the pale-yellow solid product (61% yield).

**Preparation of PAE-*r*-PEMAL.**

PAE-*r*-PHEMA was synthesized by reversible addition-fragmentation chain transfer (RAFT) random polymerization. CPDT (0.023 g, 1.0 eqv), monomer 2-azepane ethyl methacrylate (AE, 1.71 g, 120 eqv) and 2-hydroxyethyl acrylate (HEMA) (0.26 g, 30 eqv) and AIBN (4.0 mg, 0.45 eqv) were mixed in 1, 4 dioxane. After freezing and thawing for 3 times to remove resolving oxygen, the mixture was reacted at 70 °C for 12 h, and then purified by precipitating into hexane for 3 times and dried under vacuum. The product was obtained as a yellow solid (53% yield). To conjugate 1-MT and LA to PAE-*r*-PHEMA, poly(2-azepane ethyl methacrylate)-*random*-poly(2-hydroxyethyl methacrylate) ($PAE_{70}$-*r*-$PHEMA_{22}$) (0.23 g, 1 eqv), 1-MT-Boc (0.137 g, 30 eqv), 1-ethyl-3-(3-dimethylaminopropyl) carbodiimide hydrochloride (EDC·HCl) (0.16 g, 60 eqv), 4-dimethylaminopyridine (DMAP) (0.035g, 20 eqv) and LA (0.016 g, 6 eqv) were mixed in the dichloromethane. The mixture was reacted for 48 h. The polymer poly(2-azepane ethyl methacrylate)-*random*-poly(1-methyl-tryptophan co lipoic acid methyl acrylate) ($PAE_{70}$-*r*-$PEMAL_2/MT_{15}$) was further purified by dialysis in DMF to remove free LA, 1-MT-Boc and other impurities. The product obtained as a yellow solid after drying under vacuum overnight (93% yield). Poly(2-cyclohexylethyl methacrylate)-*random*-poly(1-methyl-tryptophan-co-lipoic acid methyl acrylate) ($PCM_{65}$-*r*-$PEMAL_3/MT_{17}$) polymer was synthesized using similar procedures with pH insensitive polymer $PCM_{65}$-*r*-$PHEMA_{25}$ (89% yield).

**Preparation of mPEG-*b*-PAE and mPEG-*b*-PCMA block copolymers.**

mPEG-*b*-PAE diblock copolymers was synthesized by RAFT polymerization of AE monomer using mPEG-based macro chain transfer agent (mPEG-CPAD, M*n* = 5000).[1] mPEG-CPAD was synthesized by esterification reaction between mPEG-OH and 4-cyanopentanoic acid dithiobenzoate (4-CPAD). Typically, mPEG-CPAD (0.25 g, 1.0 eqv), monomer AE (1.53 g, 130 eqv), and AIBN (4.0 mg, 0.42 eqv) were dissolved in anhydrous dioxane. After freezing and thawing for 3 times, the mixture was reacted at 70 °C for 12 h. The resulting copolymer was purified by precipitation in hexane and dried under vacuum (57% Yield). Methoxyl poly(ethylene glycol)-*b*-(2-cyclohexylethyl methacrylate) (mPEG-*b*-PCM) were synthesized by RAFT polymerization by substituting AE with 2-cyclohexylethyl methacrylate (CM) with similar procedures (49% yield).

**Preparation of 5 nm CuS nanoparticles.**

Small-sized CuS nanoparticles (5 nm) was prepared according to previous report.[2] After purifying by precipitating in acetone, the OAm modified CuS nanoparticles were stored in chloroform for next use. PAE-*r*-PEMAL/MT or PCM-*r*-PEMAL/MT polymers were further modified on the surface of CuS nanoparticles by ligand exchange procedure (denoted as $CuS_5$) at 1:1 mass ratio between CuS nanoparticles and relative polymer. The $CuS_5$ was dispersed in THF for further use.

**Preparation of PRNs$^{MT}$ and NRNs$^{MT}$.**

Programmed raspberry-structured nanoadjuvant (defined as PRN$^{MT}$) containing $CuS_5$ and mPEG-*b*-PAE polymer were prepared. Typically, mass ratio of 3:1 between $CuS_5$ and mPEG-*b*-PAE was resolved in 1 mL THF followed by addition of 5 mL Milli-Q water by syringe pump at a rate of 10 mL/h. The resulting PRN$^{MT}$ was dialyzed in water for removing THF. Similar method was applied for NRN$^{MT}$ preparation. PRN and NRN were prepared by the $CuS_5$ and mPEG-*b*-PAE or mPEG-*b*-PCM with similar procedures.

**Release Profile of 1-MT.**

The release profile of 1-MT prodrug from the PRN$^{MT}$ were determined by dialysis method. Briefly, PRN$^{MT}$ (1 mL) were transferred into a membrane tube, and 15 mL of phosphate buffer (pH 6.7) was used as the media. The samples were incubated at 37 ℃ after irradiation for 5 min or not and were incubated at 37 °C. The media (1 mL) was moved out and the fresh media was added in the designed time intervals. Subsequently, the amount of 1-MT prodrug released from the PRN$^{MT}$ suspension was characterized by UV spectrophotometer (Abs at 280 nm).

**Photothermal conversion of PRNs.**

The photothermal conversion efficiency was calculated by the formula below. Detailed calculation was given as following:

$$\eta = \frac{hs(T_{max,NP} - T_{surr}) - Q_{dis}}{I(1-10^{-A_{1064}})} \quad \text{(Eq. 1)}$$

*h* is the heat transfer coefficient, *s* is the surface area of the container, and the value of *hs* is obtained from the Eq. 4 and Figure S8. $T_{max}$ is maximum steady temperature of the solution of the PRN$^{MT}$ and $T_{Surr}$ is environmental temperature. $(T_{Max,NP} - T_{Surr})$ is the temperature change of the

solution of the $PRN^{MT}$. *I* is the laser power. $A_{1064}$ is the absorbance of the $PRN^{MT}$ at 1064 nm. $Q_{dis}$ expresses heat dissipation from the light absorbed by the solvent and container.

$$Q_{dis} = hs(T_{max,\,H_2O} - T_{surr})$$

$$\theta = \frac{T - T_{surr}}{T_{max} - T_{surr}} \quad \text{(Eq. 2)}$$

A sample system time constant $\tau_s$ can be calculated as Eq. 3.

$$t = -\tau_s \ln(\theta) \qquad \text{(Eq. 3)}$$

In order to gain *hs*, a dimensionless parameter is introduced as followed:

$$hs = \frac{m_D c_D}{\tau_s} \quad \text{(Eq. 4)}$$

**Diffusion of PRNs and NRNs at pH 6.7**

Agarose gel model was established by agarose (3%, wt%) to simulate the ECM barrier in tumor. A groove was excavated at the central site of gel. After incubation in PB buffer (pH 6.7), PRNs and NRNs were added in the groove and incubated for 24 h in moist environment to avoid drying up of gel. The resulted gel was photographed for evaluating the diffusion performance.

**Photothermal performance of PRNs and NRNs.**

PRNs and NRNs (100 μg/mL of CuS concentration calculated by ICP-MS) were irradiated with second near infrared laser-1064 nm (NIR-II) (IR1, New Industries Optoelectronics, Changchun, China) for 5 min (power density of 1 $W/cm^2$). Meanwhile, photothermal images were recorded by infrared camera (ICI7320, Infrared Camera Inc., Beaumont, Texas, USA) and analyzed using IR Flash thermal imaging analysis software (Infrared Cameras Inc.).

**Cell culture.**

Murine breast cancer 4T1 and luciferase expressed 4T1 cell (Luc-4T1) line were obtained from American Type Culture Collection (ATCC) and cultured in a humidified atmosphere at 37 °C with 5% $CO_2$. The cells were cultured in DMEM culture medium (Gibco®, Thermo Fisher Scientific, Grand Island, NY), supplemented with 10% fetal bovine serum (FBS, HyClone, Logan, UT) and 1% penicillin/streptomycin (Gibco®, Life Technologies, Grand Island, NY).

**MTT assay for PRNs cytotoxicity and their photothermal cytotoxicity evaluation *in vitro.***

MTT assays were used to evaluate the cytotoxicity of PRNs and NRNs. 4T1 cells were seeded on 96-well plates with a density of $5 \times 10^3$ cells per well. After incubating for 12 h, various

concentrations of PRNs and NRNs were added into 96-well plates. The cells were irradiated by NIR-II laser (1 W/cm$^2$) or not. After 24 h incubation, the cells were added with 5 mg/mL MTT and incubated for 4 h. Discarding the old medium, 100 μL DMSO was added. The absorbance was measured at 490 nm on a microplate reader.

***In vitro* cytotoxicity assay of PRNs evaluated by dead and live staining.**

To evaluate the photothermal cytotoxicity of PRNs on 4T1 cells, we further visualized the cell condition after 1064 nm laser irradiation (1 W/cm$^2$). 4T1 cells were seeded on 24-well plates with a density of 5 × 10$^4$ cells per well. After incubating for 12 h, various formulations were added into 24-well plates for 4 h incubation. The cells were irradiated by NIR-II laser for 5 min or not and further be stained by Calcein-AM/PI after 4 h (the dilution ratio of Calcein-AM/PI is 1:500).

**Colocalization of rab11 and PRNs.**

4T1 cells were seeded on coverslips in a 24-well plate overnight (5 × 10$^4$ cells per well). Cells were then incubated with fresh medium containing PRNs at 37 °C. After incubation for 4 h, the cells were fixed with 4% formaldehyde at room temperature for 15 min and washed for 3 times. Cells were stained with rab11 rabbit monoclonal antibody (dilution 1:100) (Cell Signaling Technology, MA, USA) at 4 °C for 12 h and followed with goat anti-rabbit FITC conjugated IgG-HRP (Beyotime Biotechnology, Jiangsu, China) for 90 min incubation to visualize the rab11. Cell nuclei were stained with DAPI.

**Transcytosis of PRNs from adhered cells to suspending cells.**

4T1 cells (5 × 10$^4$ cells per well) were cultured in a confocal dish for 12 h. At pH 6.7, PRNs/Cy5 and NRNs/Cy5 were added and incubated for 4 h, following with Hoechest 33342 addition, respectively. The dishes were washed with PBS for three times, and then 1 mL of fresh culture medium containing fresh 2 × 10$^4$ suspended 4T1 cells pretreated with Hoechest 33342 was added. The cells were under real time observation using a laser confocal scanning microscope (CLSM, Nikon ECLIPSE Ti2, Tokyo, Japan) nd the time-lapsed images were recorded.

**4T1 multicellular spheroids.**

In detail, 5 mL 1.5% hot agarose solution was coated T25 flask and cooled to room temperature naturally. 4T1 cells (2 × 10$^6$) were added to the flask and incubated with 10 mL DMEM medium for 6 days to grow into spheroids. The spheroids were transferred to 6-well

plates and incubated at pH 6.7 or pH 7.4 DMEM medium. Meanwhile, the spheroids were incubated with PRNs/Cy5 and NRNs/Cy5 (100 μg/mL, CuS concentration calculated by ICP-MS30020) for 4 h. After collecting and washing with PBS for 3 times. The spheroids were observed with CLSM Z stack method with 405 and 640 nm wavelength channels.

**Tumor penetration of PRNs.**

For real-time visualization, 4T1 tumor bearing nude mice were *i.v* injected with PRNs /Cy5 (equivalent dose of 15 mg /kg body weight). Mice were anaesthetized with 2.5% isoflurane (Linuo Pharm., Shandong, China) by a Matrx VMR tabletop with an isoflurane well-fill vaporizer (Midmark Corp., Dayton, OH, USA). To expose tumor to be visualized in the microscopy, we incised the subcutaneous tumor in an arc-shape and elevated the skin flap without injuring the feeding vessels. The coverslip was attached with the tumor surface carefully without injuring vessels. The Cy5 signals were detected using 630/670 nm excitation/emission filters. For tumor tissue sections observation, 4T1 tumor bearing nude mice were *i.v* injected with PRNs/Cy5 (equivalent dose of 15 mg /kg body weight) and were sacrificed after 12 h for frozen sections. The tumor tissues were labeled with CD31-FITC antibody (Cell Signaling Technology, MA, dilution ratio of 1:200) for tumor vessel visualization.

**Detection of calreticulin (CALR).**

4T1 cells ($5 \times 10^4$) were seeded in a 24-well plate on coverslips overnight for 12 h, and then incubated with different formulations for 4 h, following 1064 nm laser irradiation (1.0 W/cm$^2$, 5 min) or not. Following a further incubation of 4 h, CRT exposure on the surface of 4T1 cells after different treatments were detected by flow cytometry using FITC conjugated antibody to CALR (1:100, Bioss, Beijing, China).

**Detection of high mobility group protein 1 (HMGB1).**

4T1 cells ($5 \times 10^4$) were seeded in a 24-well plate on coverslips overnight and incubated for 12 h, and then treated with different formulations for 4 h, following 1064 nm laser irradiation (1.0 W/cm$^2$, 5 min) or not. Following a further incubation of 12 h, cells were incubated with individual primary antibodies against HMGB1 antibody (Beyotime Biotechnology) for overnight at 4 °C, and followed washed three times with PBS, and then the HMGB1 and cell nuclei were labelled by Alexa Fluor 647-labeled goat anti-mouse IgG (Santa Cruz Biotechnology, TX, USA) and DAPI. Coverslips were mounted on glass microscope slides with a drop of anti-fade mounting media, and then detected by CLSM.

**Detection of DC maturation.**

Murine bone marrow derived dendritic cells (BMDCs) were propagated from the bone marrow of 8-week-old Balb/c mice. BMDCs developed within clusters of proliferating cells after repeatedly addition of mouse granulocyte/macrophage colony-stimulating factor (GM-CSF) and interleukin-4 (IL-4) at a final concentration of 20 ng/mL and 10 ng/mL to the cultures, respectively. 4T1 cells were pretreated with different formulations for 4 h, following 1064 nm laser irradiation (1.0 W/cm$^2$, 5 min) or not. After a further incubation of 12 h, the dying cell (1 × 10$^5$) were collected and co-cultured with BMDCs (1 × 10$^6$) for 12 h. The cells were stained with cocktails antibodies to FITC anti-mouse CD45.2 (1:200, Biolegend, San Diego, CA, USA), PE-Cy7 anti-mouse CD11c (1:200, Biolegend), PE anti-mouse CD40 (1:200, Biolegend), PerCP-Cy5.5 anti-mouse CD80 (1:200, Biolegend), and APC anti-mouse CD86 (1:200, Biolegend), and measured by flow cytometry (BD Bioscience, Franklin Lakes, NJ, USA), and analyzed using FlowJo V10 software (Tree Star Inc., Ashland, OR, USA).

***In vitro* photothermal cytotoxic efficiency of PRNs.**

4T1 (5 × 10$^3$ per well) cells were seeded on 96-well plates and incubated for 24 h. 4T1 cells were treated with PRNs and NRNs with varying concentrations for 4 h. The fresh medium was then added to replace the old one. The NIR-II 1064 nm laser was applied to irradiate the treated cells for 5 min (1 W/cm$^2$). The cells were added with 5 mg/mL MTT and incubated for 4 h. Discarding the old medium, 100 μL DMSO was added to every well. The absorbance was measured at 490 nm on a microplate reader.

***In vivo* photothermal imaging of PRNs and NRNs.**

To evaluate the photothermal efficiency of PRNs, NRNs and $CuS_5$ *in vivo*; PRNs and NRNs (dose of 20 mg CuS per kg mouse weight, concentration of CuS was calculated by ICP-MS) were intravenously injected. 12 h later, 4T1 tumor-bearing mice were irradiated on the tumor region with 1064 nm for 5 min (power density of 1 W/cm$^2$). Meanwhile, photothermal images are captured by infrared camera and analyzed using IR Flash thermal imaging analysis software.

**Tumor-bearing animal models.**

All experiments in live animals are performed according to the relevant laws and institutional guidelines, and approved by animal ethics committee of University of Science and Technology of China (No. USTCACUC1801062). Balb/c mice were purchased from Beijing Vital River Laboratory Animal Technology Co., Ltd. To evaluate tumor suppression

experiments of PRNs and NRNs, 1 × $10^6$ 4T1 cells are subcutaneously injected into right flank of female Balb/c mice.

**Detection of ICD *in vivo*.**

For CALR staining, tumors were excised after 4 h post irradiation. Tumor tissue sections were incubated with individual primary antibodies against CALR (Cell Signaling Technology, MA) for 12 h at 4 $^{o}$C and labeled by goat anti-rabbit FITC conjugated IgG-HRP (1:100, Santa Cruz Biotechnology, Dallas, TX). For HMGB1 staining, tumors were excised after 20 h post irradiation. Tumor tissue sections were incubated with individual primary antibodies against HMGB1 (1:100, Beyotime Biotechnology, Shanghai, China) and then labeled by goat anti-rabbit Cy5 conjugated IgG-HRP. The tissues were observed by CLSM after labelling cell nuclei with DAPI.

***In vivo* photothermal evaluation and antitumor efficacy of PRNs$^{MT}$.**

4T1 tumor-bearing mice are divided into for five groups (PBS, NRNs, PRNs, NRNs$^{MT}$, and PRNs$^{MT}$). When the tumor volumes reached 100 $mm^3$, mice were injected with different formulations respectively (n = 5). After 12 h, tumors were irradiated with 1064 nm laser until the temperature are stable with a power density of 1 $W/cm^2$ for 5 min. Tumor volumes and mice weight were monitored every two days. The tumor volumes were measured using calipers and calculated according to the reported equation: Volume = (Tumor length) × $(\text{Tumor width})^2 \times 0.5$.

**Flow cytometry assay of cell surface markers.**

After 10 days from first tumor treatment, the mice were sacrificed and spleens were excised. The spleen pieces obtained for single-cell analysis were gently meshed though nylon mesh. Red blood cells were lysed by Ack lysing buffer (Biosharp, Hefei, China) according to the manufacturer’s instructions. Cell preparations were stained for 30 min at 4°C with cocktails containing combinations of fluorochrome conjugated monoclonal antibodies for cell surface markers (FITC anti-mouse CD45.2 (1:200, Biolegend), APC-Cy7 anti-mouse CD3 (1:200, Biolegend), APC anti-mouse CD4 (1:200, Biolegend), PE-Cy7 anti-mouse CD8α (1:200, Biolegend), PerCP-Cy5.5 anti-mouse CD44 (1:200, Biolegend), and PE anti-mouse CD62L (1:200, Biolegend). In order to analyze Treg cells and CD8+ T cells in tumor tissues, we stained the cells according to manufacturer’s protocols (anti-rabbit CD8+ antibody, CST, 1:200; anti-rabbit Foxp3+ antibody, CST, 1:200).

**Immunohistochemical and immunofluorescence analyses.**

At the end of therapy, the tumor tissues excised from sacrificed mice were fixed in 4% formaldehyde for 24 h and embedded in paraffin. The tumor tissues slices were observed after H&E staining. For immunofluorescence staining, tumors were excised for frozen section at $10^{th}$ day post-irradiation treatment.

**Suppression on 4T1 metastatic tumor model by a combination of $PRNs^{MT}$ and α-PD1.**

4T1 tumor-bearing mice are divided into six groups (PBS, NRNs, PRNs, $NRNs^{MT}$, $PRNs^{MT}$ and a combination of $PRNs^{MT}$ and α-PD1). After 12 h post injection of various formulations, tumors were irradiated with 1064 nm laser for 5 min with a power density of 1 $W/cm^2$ (n = 8). After 24 h, all mice were *i.v.* injected with Luc-4T1 ($5 \times 10^4$ per mice) and tumor volume was monitored every two days. Xenogen IVIS Lumina system was used to monitor cancer metastasis of mice at day 12, 18 and 24 post-injections of Luc-4T1 cells. Before bioluminescence imaging, relevant Luciferin substrate was intraperitoneally injected. When mice tumor volume reached to 2000 $mm^3$ or died, mice were sacrificed and lungs were excised and photographed. The pictures of pulmonary nodules were captured by camera and the number of nodules was counted.

Statistical Analysis.

Data are expressed as the mean ± S.D. Statistical analysis between groups was performed using GraphPad Prism 8.0. For data with multiple groups, the statistically significant differences were assessed using one-way analysis of variance (ANOVA) or two-way ANOVA with Tukey's multiple comparisons test. For comparisons between two groups, unpaired two-tailed Student's t-tests were used. The sample sizes (biological replicates), specific statistical tests, and the main effects of our statistical analyses for each experiment are detailed in each figure legend. Significant difference among group were assigned as *$p<0.05$, **$p<0.01$and ***$p<0.001$, respectively. *$p<0.05$ was considered statistically significant in all analyses (95% confidence level).

Reference

1. Wang, Y., Zhou, K., Huang, Hensley, C., Huang, X., Ma, X., Zhao, T., Sumer, B., DeBerardinis, R., Gao, J. *Nat. Mater.* **2014**, *13*, 204–212.

2. Mou, J., Li, P., Liu, C., Xu, H., Song, L., Wang, J., Zhang, K., Chen, Y., Shi, J. and Chen, H. *Small*, **2015**, *11*, 2275-2283.

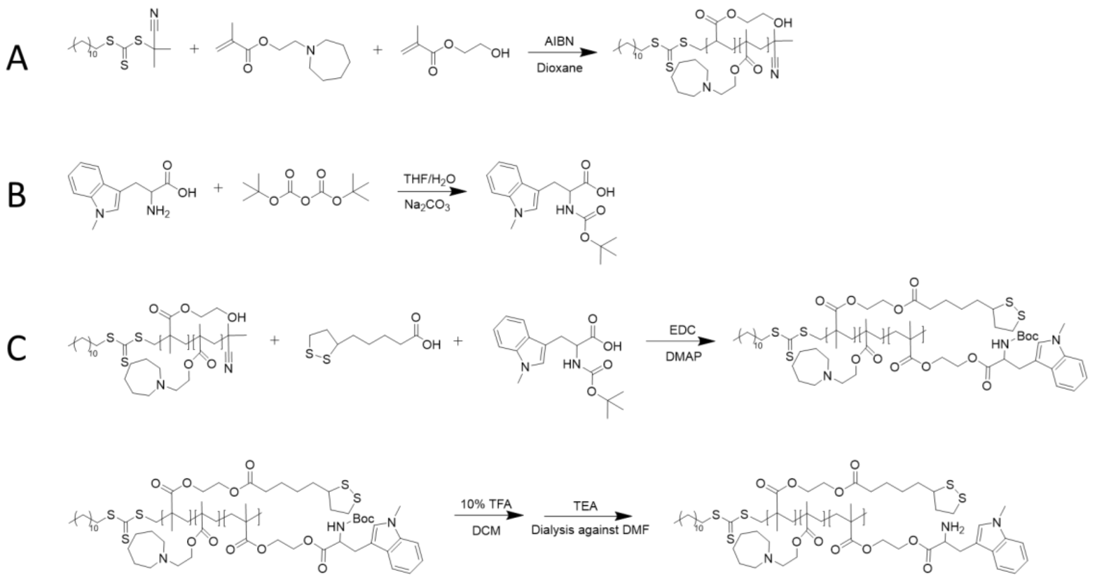


**Figure S1.** Synthetic routes of pH-responsive polymer. (A) synthetic routes of PAE. (B) Synthetic routes of 1-MT-Boc. (C) Synthetic routes of PAE-*r*-PEMAL/MT.

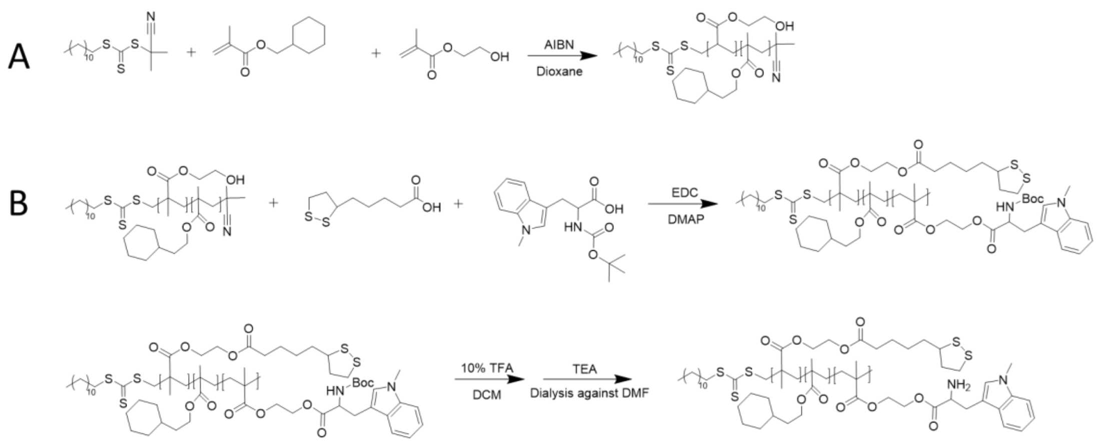


**Figure S2.** Synthetic routes of non-pH sensitive polymer. (A) The synthetic routes of PCM-r-PEMAL. (B) The synthetic routes of PCM-r-PEMAL/MT.

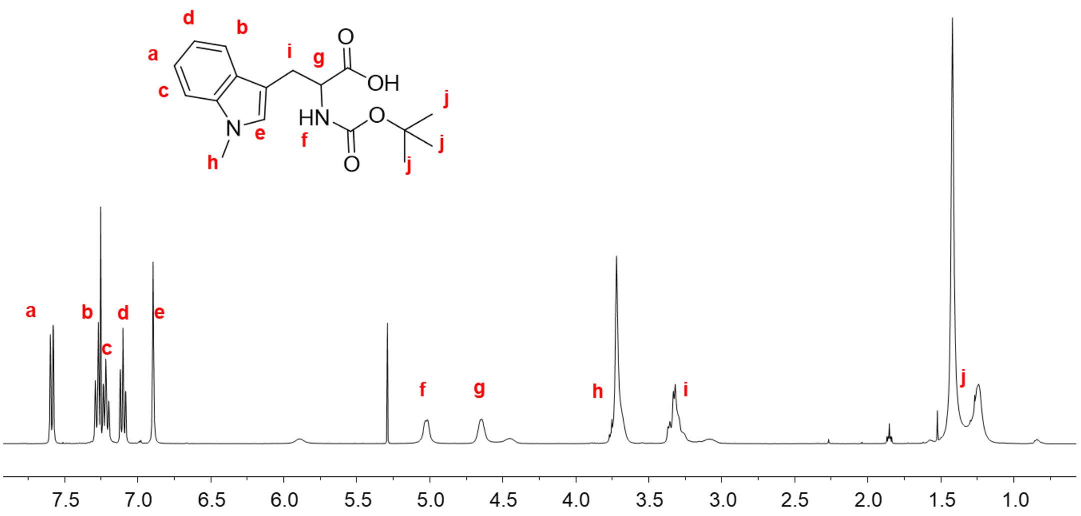


**Figure S3**. $^{1}$H NMR spectra of 1-MT-Boc.

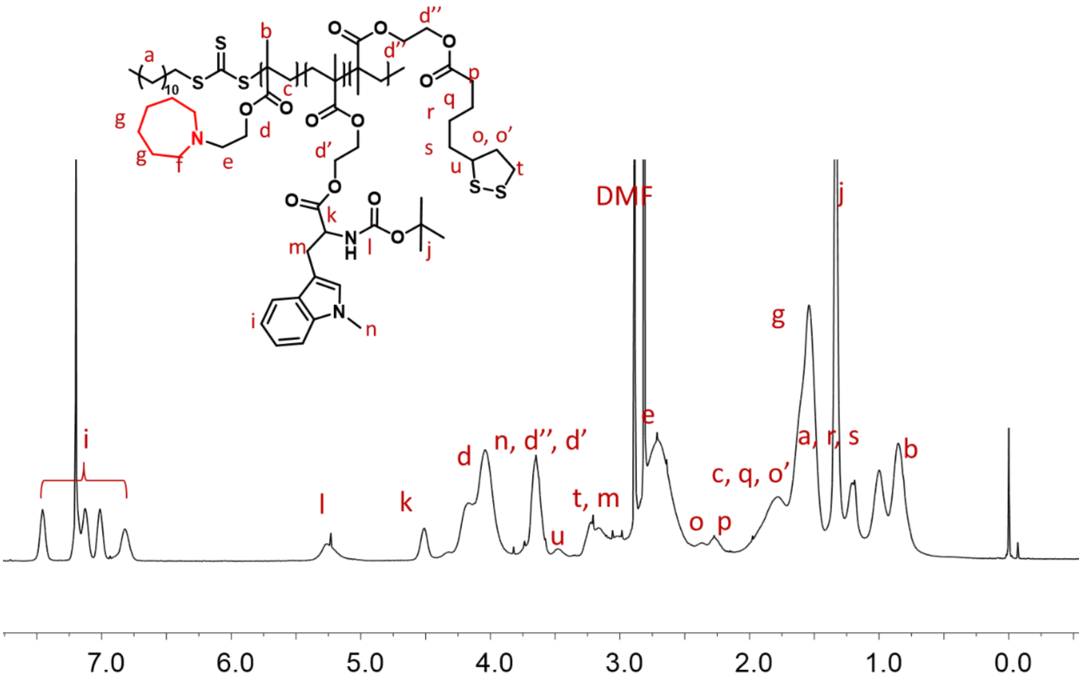


**Figure S4**. $^{1}$H NMR spectra of PCM-*r*-(EMAL-MT)-Boc.

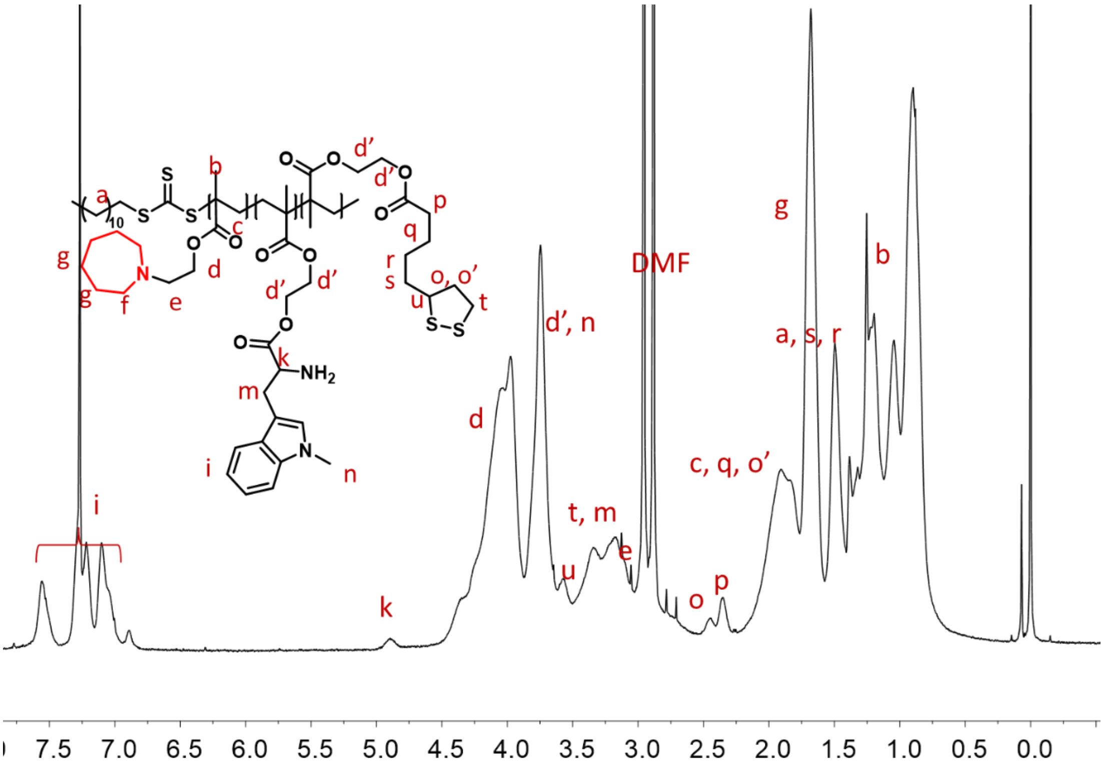


**Figure S5**. $^{1}H$ NMR spectra of PAE$_{70}$-*r*-PEMAL$_{2}$/MT$_{15}$.

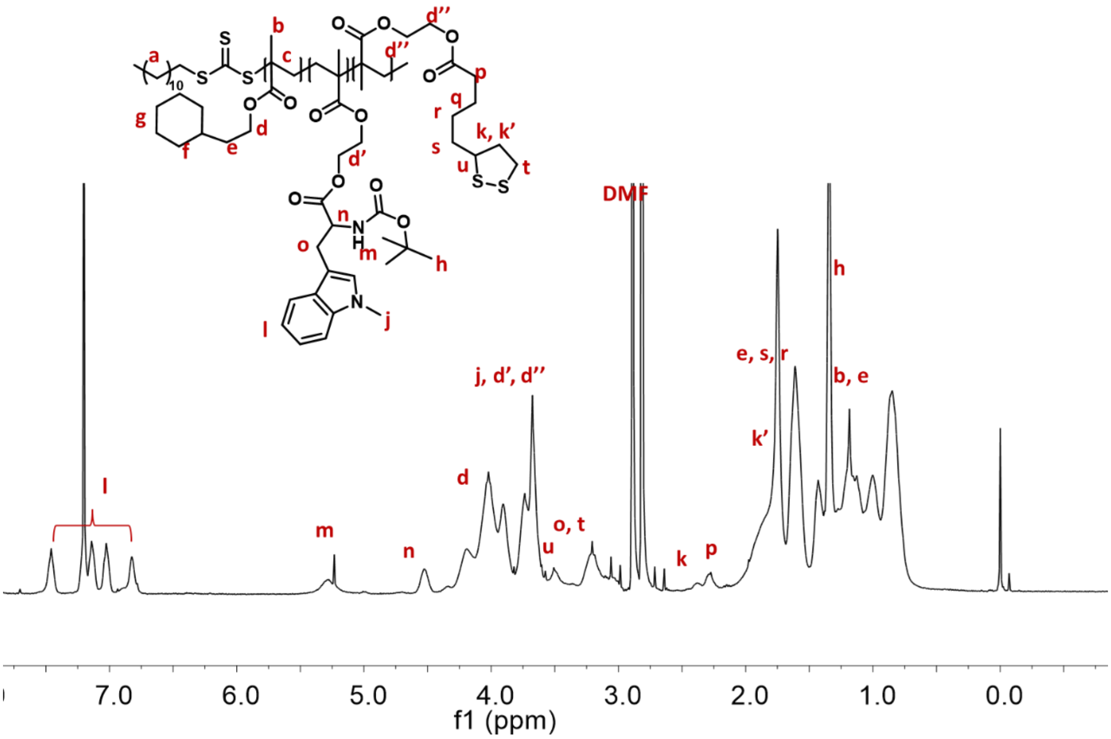


**Figure S6**. $^{1}$H NMR spectra of PCM-MT-Boc.

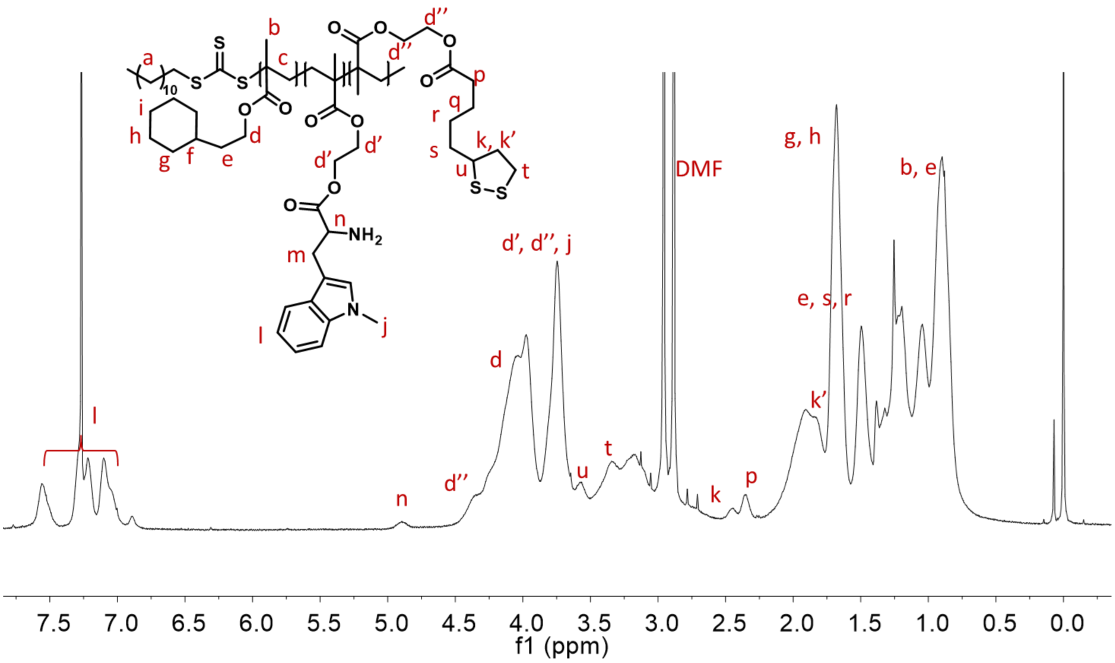


**Figure S7**. $^{1}$H NMR spectra of $PCM_{65}$-*r*-$PEMAL_3/MT_{17}$.

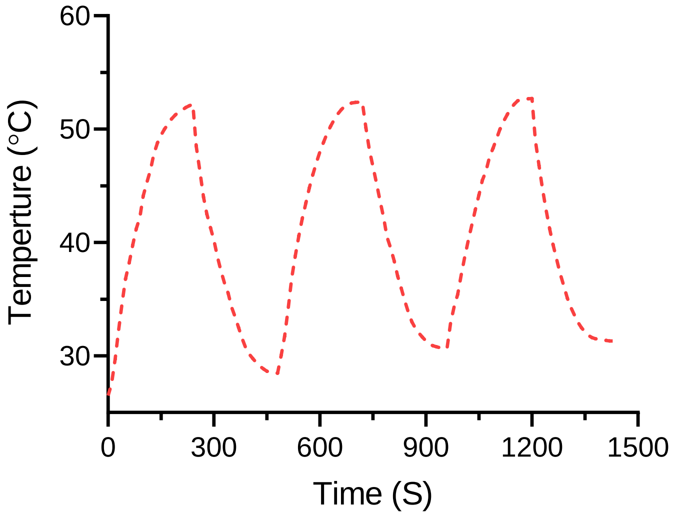


**Figure S8**. Time-dependent temperature changes of PRNs under 3 heating-cooling cycles (1 W/$cm^2$).

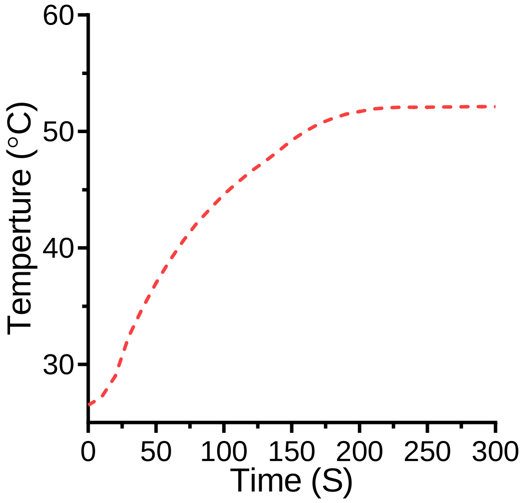


**Figure S9**. Time dependent temperature development of PRNs at pH 6.7 (1 W/cm$^2$).

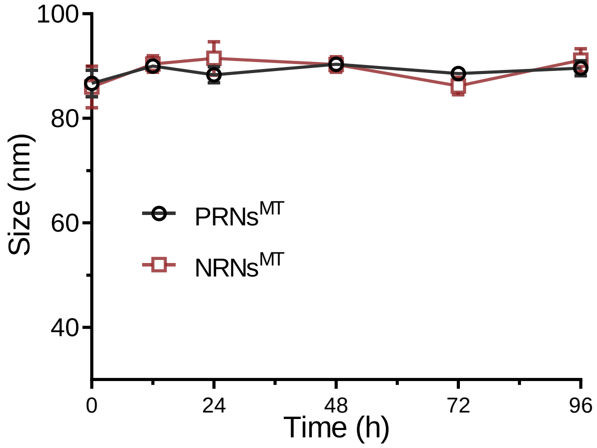


**Figure S10**. Time-dependent size changes of PRNs$^{MT}$ and NRNs$^{MT}$ in pure water.

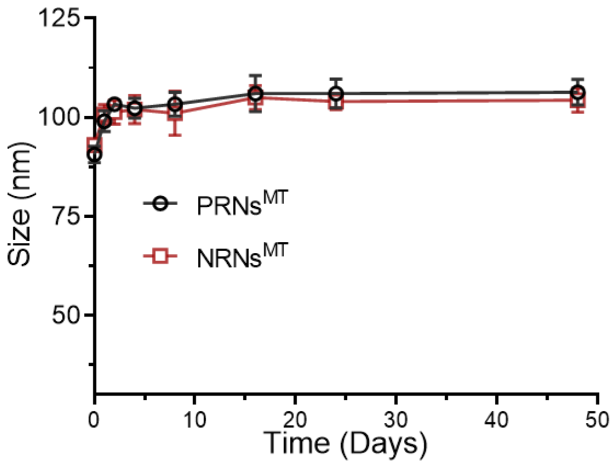


**Figure S11.** Time-dependent size changes of PRNs$^{MT}$ and NRNs$^{MT}$ in 10% FBS.

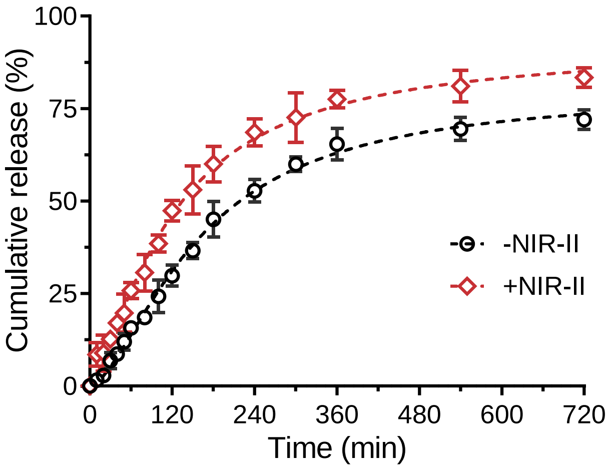


**Figure S12**. Cumulative release of 1-MT with or without 1064 nm light irradiation for 5 min at beginning of assay (pH 6.7).

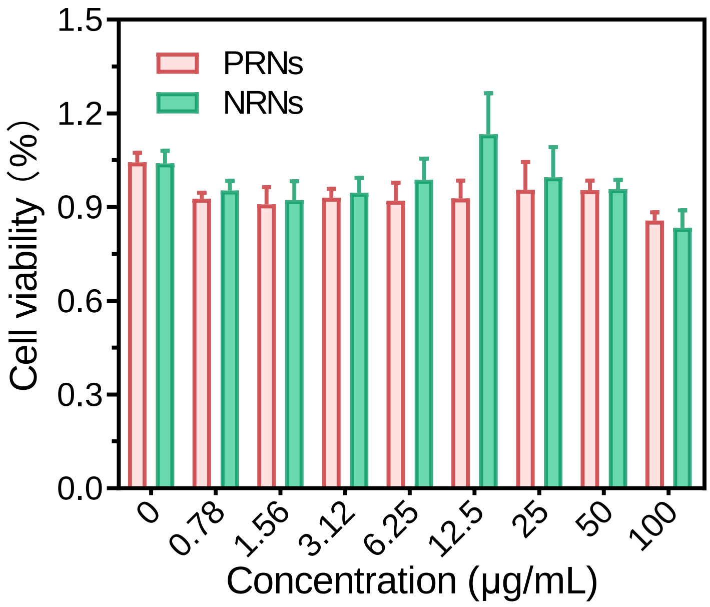


**Figure S13**. MTT assay for evaluating the cytotoxicity of PRNs and NRNs.

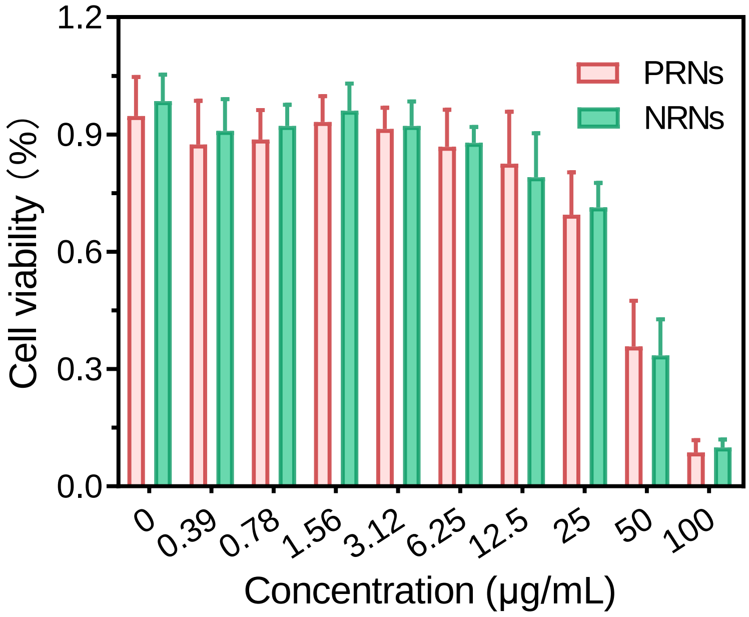


**Figure S14**. MTT assay for evaluating the cytotoxicity of PRNs and NRNs under NIR-II laser irradiation for 5 min.

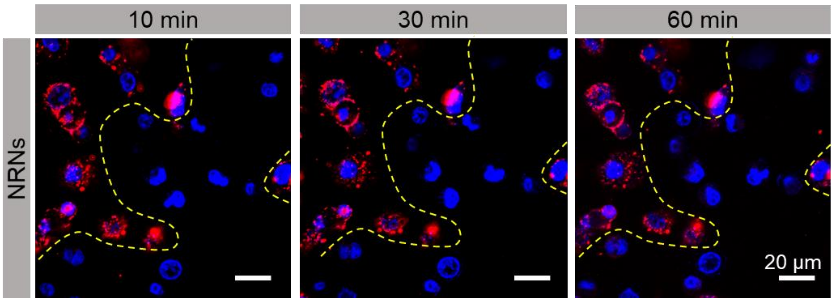


**Figure S15**. Real time confocal images of the transcytosis of NRNs observed by CLSM.

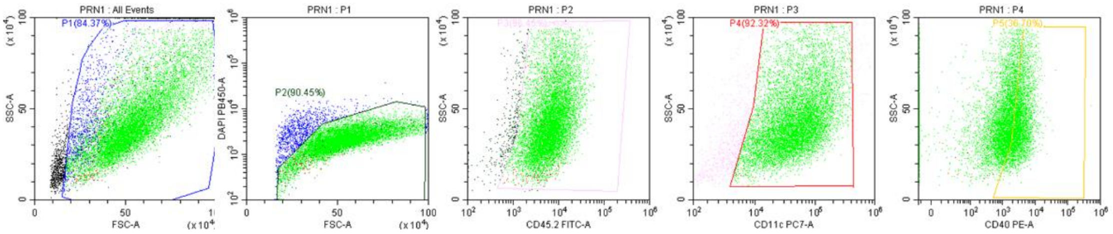

**Figure S16**. Gating strategy for flow cytometric analysis of extracellular makers on BMDCs after treatment with PRNs plus NIR-II laser irrdiation.

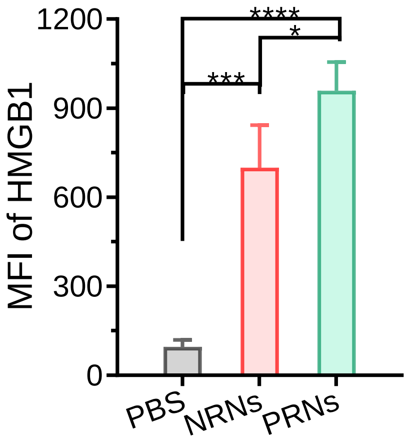


**Figure S17**. Corresponding quantifications of the MFI of HMGB1 of 4T1 tumors.

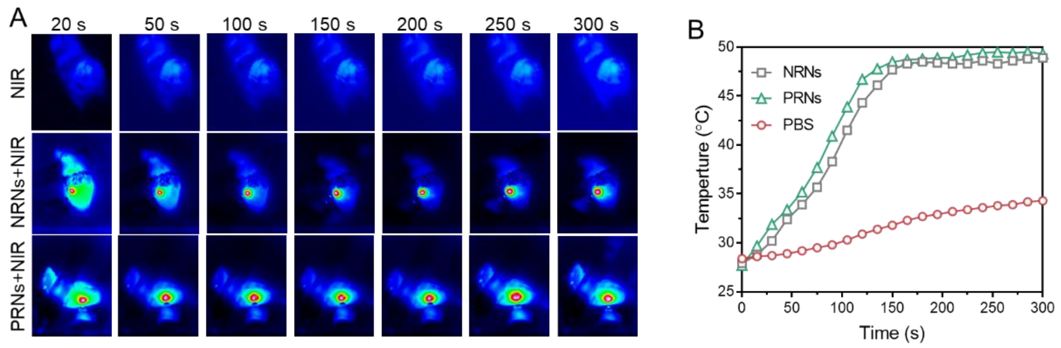


**Figure S18**. (A) Time dependent infrared thermal images of 4T1 tumor bearing mice treated with PBS, NRNs and PRNs under 1064 nm laser irradiation. (B) Tumor temperature changes as a function of irradiation time. Data obtained from panel A.

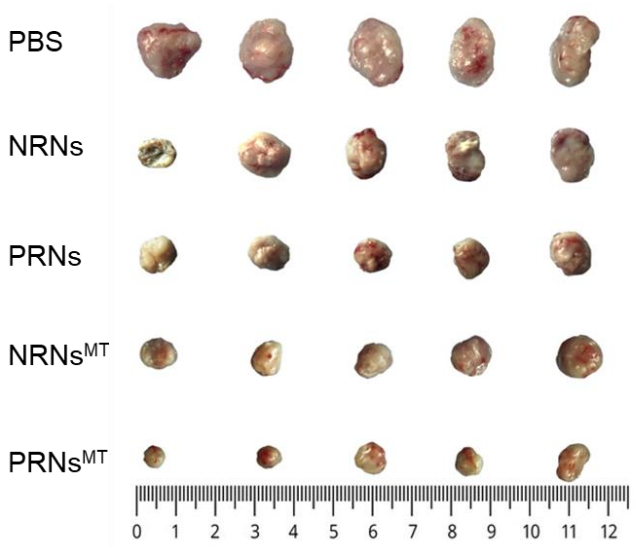


**Figure S19**. Photographs of excised tumors after from mice.

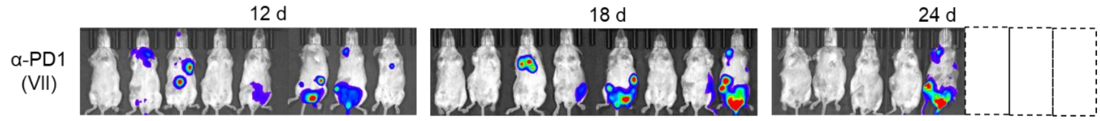


**Figure S20**. IVIS images of metastatic Luc-4T1 tumor after treating with α-PD1.

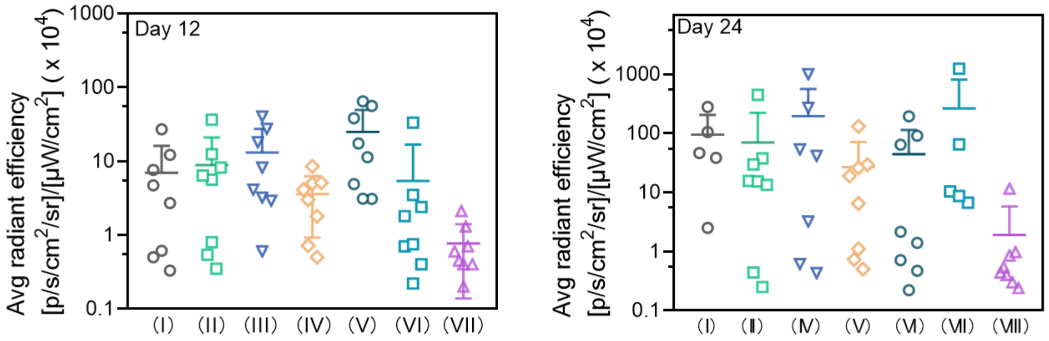


**Figure S21**. Corresponding quantification of bioluminescence signal in mice at day 12 and day 24.

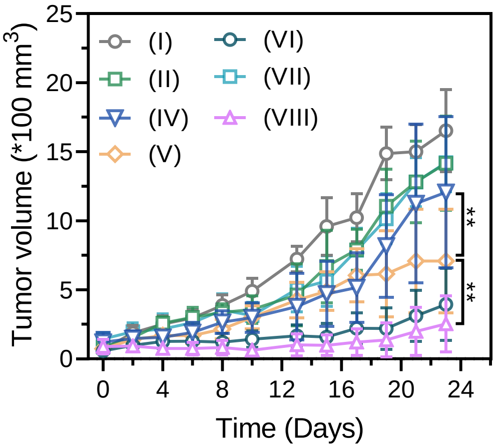


**Figure S22**. Time-dependent primary tumor size development of mice after *i.v.* injection of luc-4T1 cells and PTT treatment on primary tumor.

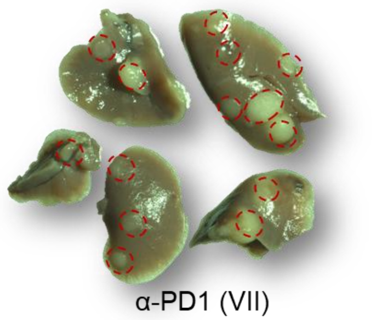


**Figure S23**. Representative photographs of lung lobes of mice with α-PD1 treatment as viewed by stereo microscope scan (the red circles indicated metastatic nodules).
